# Engineering Exosomes for CNS Disorders: Advances, Challenges, and Therapeutic Potential

**DOI:** 10.3390/ijms26073137

**Published:** 2025-03-28

**Authors:** Eun Chae Lee, Dongsic Choi, Dong-Hun Lee, Jae Sang Oh

**Affiliations:** 1 Department of Medical Sciences, Graduate School, The Catholic University of Korea, Seoul 06591, Republic of Korea; lec9589@catholic.ac.kr; 2 Department of Biochemistry, Soonchunhyang University College of Medicine, Cheonan 31151, Republic of Korea; dongsic@sch.ac.kr; 3 Department of Neurosurgery, Uijeongbu St. Mary’s Hospital, College of Medicine, The Catholic University of Korea, Seoul 11765, Republic of Korea

**Keywords:** exosome, nanoparticle, central nervous system, engineering exosome

## Abstract

The development of targeted drugs for diseases of the central nervous system (CNS) is a significant challenge due to the structural complexity and functional specificities of these systems. Recently, exosomes have emerged as a promising therapeutic platform, given their unique capacity to traverse the blood-brain barrier and deliver bioactive molecules to target cells. This review examines recent advances in exosome research with a particular focus on CNS diseases, emphasizing their role as carriers of therapeutic cargo, including proteins, RNAs, and lipids. Nevertheless, significant challenges remain before exosome-based therapies can be translated from preclinical research to clinical applications. These include the need for scalable production and standardized isolation methods. Despite these hurdles, ongoing studies continue to shed light on the mechanisms of exosome-mediated neuroprotection and neurodegeneration. This paves the way for innovative therapeutic strategies to address CNS disorders.

## 1. Introduction

Exosomes represent a specific type of extracellular vesicles (EVs) that are generated within cells and subsequently released into the extracellular space [1]. They are secreted in eukaryotic organisms for the purpose of facilitating information exchange between cells and have a size of 30–150 nanometres (nm) [2,3]. In addition to exosomes, EVs released outside the cell include microvesicles (MVs) and apoptotic bodies [4,5]. These have different sizes, structural diversity, characteristic contents, and cellular origin [6]. The exchange of information between cells within a living organism is facilitated by exosomes, which contain a range of biomolecules, including proteins, nucleic acids, fats, and metabolites, that enable signal transmission between cells.

Exosomes contain a variety of bio-derived substances and reflect the approximate characteristics and status of the mother cell that secretes them. This means it is possible to diagnose diseases by detecting the presence or absence of specific biomolecules in exosomes or inject drugs or proteins into exosomes [7,8]. The inclusion of this substance could facilitate the development of a next-generation transporter for the treatment of currently incurable diseases such as cancer [9,10].

Over the past decade, there has been a significant increase in research activity surrounding the use of nanoparticles in drug delivery systems, with the aim of enhancing the therapeutic efficacy of chemical and biomolecular drugs [11]. Nevertheless, these systems have not been able to address various issues, including cytotoxicity, rapid degradation, and the need for a more precise synthesis process tailored to the specific characteristics of the disease, from a clinical perspective. Furthermore, only a limited number of systems have received Food and Drug Administration (FDA) approval [12,13]. The most popular types of nanoparticle treatments approved by the FDA are those using polyethylene glycol (PEG) [13,14]. To overcome these limitations, research on nano-drug delivery platforms using exosomes, which have natural biocompatibility, has recently been actively conducted [15]. Exosomes are 50–150 nm endoplasmic reticulum released from cells, with different features such as their small size, lack of immunogenicity, the ability to escape from the endosomal pathway, and their ability to enhance their phospholipid bilayer membrane enhancing their ability to deliver cargoes directly into the cytoplasm make exosomes an appropriate drug delivery vehicle.

Cranial nerve disorders/diseases are one of the leading causes of sequelae and death worldwide [16]. In order to solve these problems, research demands for various treatments and rehabilitation are increasing. However, drug development is the most difficult field because of the blood-brain barrier (BBB), which prevents most drugs currently under development from entering the brain parenchyma [17,18,19]. The BBB functions as a tight barrier to protect the central nervous system (CNS) from potential neurotoxic agents and regulates the selective transport of certain molecules and nutrients to maintain CNS homeostasis. Water molecules and small ions pass through the brain capillaries through the channels, and small molecules of less than 500 kDa can cross the BBB by passive diffusion [20]. Additional studies are being conducted to overcome limitations in the ability to pass the BBB in existing research on various stem cell treatments. However, there are many limitations as a single stem cell therapy, but exosomes can overcome these problems [21,22]. It can also be conveniently delivered via intravenous or intranasal injection. In particular, miRNAs and protein-derived exosomes can be usefully used as biomarkers for early diagnosis of exosomes, disease progression, and treatment response [23,24]. However, most research fields are on tumor treatment, and research on CNS diseases is lacking [25].

Various studies are being conducted focusing on phenomena occurring in neurological diseases that are difficult to diagnose and treat using these characteristics, but they are facing various limitations in the commercialization and development stages. In fact, until recently, there has been no FDA-approved exosome product, and it is challenging in various clinical trials [26]. In this review, various exosome engineering systems and visions of the clinical application of engineered exosomes in neurological diseases are described.

## 2. Characteristics of Exosomes

Exosomes, as double-layered vesicles, play a crucial role in eliminating cellular waste while facilitating both intercellular and intracellular communication [27]. They function as biological carriers, transporting proteins, lipids, and nucleic acids, which highlights their significance in cell signaling and biomarker discovery [28]. Not only exosomes but also vesicular and non-vesicular nanoparticles may be involved in intercellular communication. Nanoparticles may also contribute to calcification of the extracellular matrix and transport of waste and/or nutrients [28]. Exosomes are attracting attention as a next-generation drug delivery platform due to their high delivery efficiency, low immunogenicity, excellent biocompatibility, and ease of passage through the blood-brain barrier [29,30]. Through either endogenous or exogenous modification, exosomes can carry cargoes with various therapeutic effects, such as small molecule drugs, miRNA, siRNA, peptides, etc. [4]. However, natural exosomes are poorly targeted and easily eliminated by the body’s immune system [31]. Due to their highly modifiable nature and biological properties, exosomes need to be engineered based on their lipid bilayer structure to add specific surface molecules (e.g., molecules targeting specific tissues or antiphagocytic proteins) [32]. This can improve the targeting and retention time of exosomes in the body [33,34]. Therapeutic molecules and molecules with specific functions can be modified in different ways in the lumen or on the surface of exosomes [35,36].

**Figure 1:**
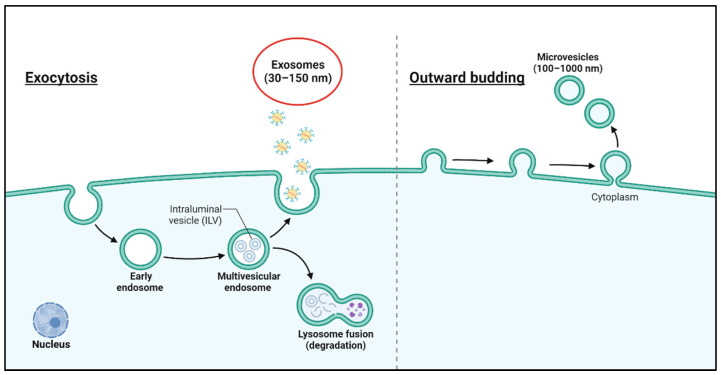
A visual representation of the process of exosome formation. The production of these membrane-bound vesicles (exosomes) occurs through a series of steps within the cell. Initially, early endosomes are formed from the cell membrane or the inner membrane structure. These early endosomes then progress through subsequent stages, ultimately transforming into a “multivesicular body” (MVB). Within the MVB, the formation of smaller vesicles occurs, which are secreted into the extracellular space as exosomes. The primary function of these exosome-derived vesicles is to facilitate signaling between cells, the transport of substances, and the execution of other physiological functions. The production of these vesicles is primarily dependent on the interaction between the cell membrane and MVBs, as well as on the presence of specific proteins.

Exosomes are produced and released by virtually all cell types, including a variety of brain cells, such as neurons, astrocytes, microglia, and oligodendrocytes. Isolation of pure exosomes involves methods such as sucrose cushions, density gradients, size exclusion chromatography, or sequential ultracentrifugation and filtration, with the resulting vesicles requiring thorough characterization for size, morphology, and biochemical properties. Since exosomes are the predominant vesicle type in preparations containing other extracellular vesicles, many authors use the terms “extracellular vesicles” (EVs) and “exosomes” interchangeably [37]. Extracellular vesicles are classified into exosomes (30–150 nm), microvesicles (200–1000 nm), and apoptotic bodies (800–5000 nm) based on their size. For simplicity, we use the term “EVs” when referencing studies that do not adhere to rigorous isolation protocols for pure exosome preparations [4]. Although exosomes are commonly referred to in many studies, it should be noted that preparations may also contain small amounts of other extracellular vesicles (Figure 1).

Exosomes are categorized into different generations based on their development and use. First-generation exosomes, derived from cells, serve as a “cell avatar”, enabling the therapeutic effects of stem cells through the substances released by exosomes [38]. Second-generation exosomes are enhanced by drug loading, providing improved therapeutic potential. Third-generation exosomes can carry high-molecular drugs, overcoming limitations of previous technologies [39]. Fourth-generation exosomes are distinguished by advanced delivery technology, allowing pharmacological substances to be delivered directly into the intended recipient cells from the medium [38].

## 3. Engineering Techniques to Enhance Exosome Function

Exosomes are materials with intrinsic biocompatibility and high physicochemical stability [40]. Nonetheless, it has been confirmed that it is rare for unengineering exosomes to reach the brain after systemic administration [41]. Therefore, in order to present exosomes as a suitable therapeutic delivery system for neurological diseases, research is needed to load effective drugs and improve targeting ability by engineering the surface and lumen [42,43]. Editing through genetic and chemical manipulation of donor cells is endogenous fertilization, while loading therapeutic drugs or cargo on the membrane is extrinsic fertilization [44,45]. Extrinsic modifications to enhance drug efficacy in an effort to overcome neurological diseases are described below [46].

### 3.1. Cargo Packaging

**Figure 2:**
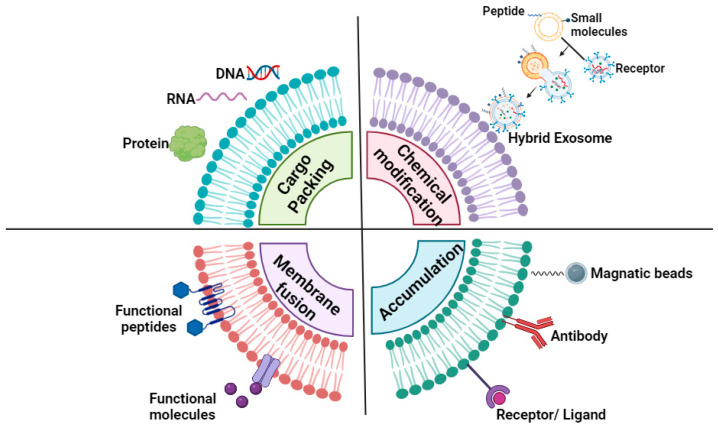
Characterization of exosome surface engineering methods for enhanced cargo delivery and targeting. The process of cargo packaging entails the encapsulation of various biomolecules (including, but not limited to, nucleic acids, proteins, and small molecules) into exosome form through natural cellular processes or artificial loading techniques. This process serves to enhance the therapeutic payload. The second method is chemical modification, where surface ligands, peptides, or antibodies are conjugated to exosome membranes, improving targeting specificity and interaction with specific cell types or tissues. Membrane fusion is a technique in which exosome membranes are fused with other vesicles or liposomes to increase their stability and payload delivery efficiency. Finally, accumulation highlights how engineered exosome accumulation at target sites can be achieved through enhanced biodistribution, prolongation of circulation time, and improvement of therapeutic efficacy.

Exosomes are defined as nanometer-sized endoplasmic reticulum composed of a double phospholipid membrane, which is similar to the structure of a cell membrane. The interior of the exosome contains proteins, nucleic acids, and carbohydrates and also includes other components, which are collectively referred to as exosome cargo [47]. Exosome cargo comprises a wide range of signaling factors, which are known to be specific to cell types and regulated differently depending on the environment of the secretory cell [48]. Exosomes are defined as intercellular signaling mediators secreted by cells. It is well documented that various cell signals are transmitted through them, regulating cell behavior, including activation, growth, migration, differentiation, dedifferentiation, apoptosis, and necrosis of intended recipient cells [49,50]. Exosomes contain specific genetic material and bioactive factors depending on the nature and state of the cell from which they are derived [51]. In the context of proliferating stem cell-derived exosome research, these membrane-bound vesicles have been shown to regulate a number of cellular processes, including cell migration, proliferation, and differentiation. Furthermore, studies have demonstrated that these exosomes reflect the characteristics of stem cells that are associated with tissue regeneration [52,53]. In a particular study, the effect of mesenchymal stem cell-derived exosome treatment on the differentiation potential was confirmed in the context of osteoarthritis [52]. In order to load the cargo into exosome form, it is necessary to bypass the exosome barrier and add exogenous drugs to donor cells. This will load the exosome in advance in situ. The co-expression of protein cargo within the donor cell has been demonstrated to bind to exosomes via protein-protein interaction [54]. Electroporation represents a well-established technique for passive packaging, whereby temporary pores are formed in the exosome membrane. The diffusion of the drug occurs through these pores, and subsequently, the integrity of the exosome membrane is restored [55]. Electroporation is a technique that has been employed to encapsulate a wide range of cargoes, including proteins and mRNAs. In the context of biopharmaceuticals, the loading of these cargoes into the exosome has been demonstrated to enhance in vivo safety, blood circulation, and cell-targeting efficiency [56] (Figure 2).

### 3.2. Chemical Modification

Chemical modification of exosomes can increase stability as well as further maximize targeting and delivery efficacy. Chemical modification involves the display of various natural or synthetic ligand receptors on the surface of exosomes through various techniques, which can be broadly categorized into covalent and non-covalent modifications. In one study, a copper-free azide–alkyne cycloaddition method was modified and applied to the surface of MSC-derived exosomes as a faster and more convenient in vivo chemical biology method. Using this modified exosome, we confirmed that it targets the ischemic brain damage area in mice with middle cerebral artery occlusion (MCAO) [57]. In the case of non-covalent association, electrostatic interactions between surface peptides and exosomes played a key role in improving cellular uptake and release of exosomes. A number of recent papers have reported on the chemical-genetic engineering of exosomes to improve the delivery of therapeutics [58]. Recently, various studies are being conducted to utilize exosomes to effectively deliver drugs to difficult-to-target areas, such as the BBB or microstructural environment, by loading them with chemotherapy agents, nucleic acid therapeutics, and peptides [20,59,60] (Figure 2).

### 3.3. Membrane Fusion

In order to change the properties of exosomes, there is great interest in new hybrid exosomes by direct membrane fusion of exosomes and synthetic liposomes [61]. Typically, exosomes deliver their molecular cargo to the cytosol via endocytosis or membrane fusion [62]. Different technological applications of exosome carriers in drug delivery systems may require modifying and tuning the exosome lipid bilayer membrane [63]. A research paper identified a novel and facile membrane engineering strategy to modify the surface of exosomes using direct membrane fusion between synthetic liposomes and exosomes after secretion from parent cells [62]. As a result of the study, the half-life of exosomes in the blood can be improved by optimizing the properties of the exosome surface, reducing immunogenicity and increasing colloidal stability (Figure 2).

### 3.4. Accumulation

Among exosome engineering methods, the magnetic bead method can recognize flags on the exosome surface to capture engineered exosomes. It is also used as an exosome extraction method but utilizes the magnetism of the extracted exosomes to recognize pH-responsive motifs [64].

In addition, one study utilized A33 antibodies to create functionalized exosomes to target colorectal cancer, demonstrating potentiometric binding and affinity to colorectal cancer cells and enhanced antiproliferative effects [65]. Another method involves attaching exosomes to the membrane of an intended recipient cell to directly manipulate the surface of the recipient cell through ligand–receptor interactions. This method is currently being actively utilized in various studies and shows promise as a cancer treatment [9,66].

In a single study, the researchers modified serum-derived exosomes with mannose with the intention of enhancing their uptake by murine dendritic cells and promoting targeted lymphatic accumulation. The mannose-modified exosomes demonstrated an increased interaction with mannose receptors on dendritic cells, which improved cellular uptake and retention in the lymphatic system. This approach has the potential to improve the targeted delivery of therapeutic agents via exosomes, particularly in immunological and inflammatory applications [67] (Figure 2).

## 4. Application of Exosomes for the Treatment of Neurological Diseases

Neurons, the most fundamental structural and functional units of the CNS, are the most important cell type in the nervous system and can receive and transmit impulses via chemical or electrical signals to peripheral and glial cells [68]. In the CNS, glial cells are represented by oligodendrocytes, astrocytes, ependymal, and microglial cells, whereas in the peripheral nervous system (PNS), they are represented by Schwann cells and satellite glial cells. Microglia, astrocytes, oligodendrocytes, and neurons can actively secrete exosomes and play an important role in cell-to-cell communication. In the CNS, exosomes can spread throughout the brain via cerebrospinal fluid (CSF) as well as short distances within cells [69]. Therefore, exosomes are greatly involved in the central nervous system and in the study of therapeutic agents for nervous system diseases from a pathological point of view [16,70]. This perspective is used in diagnostic studies of CNS diseases and is expected to be an ideal candidate for drug delivery systems (DDS) [71]. In addition, in various research papers recently, exosomes have shown remarkable efficacy in neurogenesis and repair. It is also known to mediate and improve communication within neurons [72].

In a study using exosomes engineered for stroke, there was a study showing that exosomes loaded with pigment epithelium-derived factor (PEDF) derived from adipose-derived mesenchymal stem cells (ADMSCs) had a therapeutic effect on brain damage caused by MCAO ischemic stroke animal model [73]. PEDF is a neuroprotective protein with anti-inflammatory and antioxidant properties, and research has demonstrated that when loaded onto exosomes, it participates in cell death and protects neurons through an autophagic response. In addition, studies have been conducted showing that engineering exosomes, which are a hybrid plasmid called Lamp2b-central nervous system-specific rabies viral glycoprotein (RVG) fused to exosome membrane proteins, are specifically delivered to neurons, microglia, and oligodendrocytes in the brain and help with stroke recovery at B6C3-Tg animal. Finally, miR-223 is the most abundant miRNA in exosomes released from mesenchymal stem cells (MSCs). Exosomal miR-223 has recently been confirmed to have potential as a new target treatment for stroke, with studies showing that it inhibits and alleviates the salt-induced response mediated by microglial M1 polarization (Table 1). Although various studies are underway to develop treatments using exosomes, it is necessary to distinguish and define the difference between exosomes and non-vesicular nanoparticles within nanoparticles released from cells [74] (Table 1).

In another study, engineering exosomes that labeled exosome membranes with neuropilin-1-targeted peptide (RGERPPR, RGE) to target glioma cells were confirmed to reside in tumors for a long time [75]. This engineering exosome promoted BBB penetration and accumulated heavily in glioma cells, confirming its potential as a targeted therapy when labeling a therapeutic agent.

Efficient drug delivery in neurological disease therapeutic delivery research remains a challenge. This is due to several limitations due to the existence of the BBB [76]. However, the transcytosis mechanism of exosomes crosses the BBB throughout the cell. This mechanism is mediated by the binding of ligands to specific receptors, which in turn induces receptor-mediated endocytosis and transport of the further incorporated endosome compartment to the other side of the membrane. RGE peptide is a specific ligand for neuropilin-1 (NPR-1) and has the ability to penetrate tumor tissue. NRP-1 is a protein temporarily expressed in glioma cells and tumor vascular endothelium. In this study, it was confirmed that exosomes engineered with RGE peptide using curcumin (Cur), a plant polyphenol compound with proven anti-tumor activity, promoted therapeutic effects in U251 cells [77,78]. This binding method capable of targeting the target peptide shows promising results for engineering exosomes with targeting to the cancer [79].

Considering global trends in exosome research, there is a growing emphasis on understanding exosome heterogeneity and exploring their diverse functions [80]. It is essential to develop an analytical method that will serve as a reference point for characterization [81].

Additionally, such technological development research must overcome the limitations of existing technologies. This is possible through convergence research in engineering and bio, so convergence research needs support [82].

In addition, as attempts to utilize exosomes as next-generation drug carriers and therapeutic agents continue to increase, the development of mass production and high-purity exosome purification methods is necessary [83]. The exosome origin cell line, culture and harvest conditions, isolation and purification methods, and stability should be demonstrated by analyzing harvested exosomes using analytical methods such as nanoparticle tracking analysis (NTA), nano-flow cytometry, proteomics, lipidomics, and transcriptomics [48,83,84]. Basic and clinical research is being actively conducted around the world to utilize exosomes’ natural function of carrying and delivering various bioactive substances in the body as a therapeutic agent. Research is also being actively conducted on methods for carrying various drugs (chemicals, proteins, nucleic acids, etc.) utilizing the biocompatibility of exosomes, tissue-targeting functions, and improvement of pharmacokinetics [85]. Exosomes containing cholesterol-modified AMO181a were being investigated as a novel nanocarrier to cross the BBB to reduce cerebral cell death and neurorecovery and were brain-deliverable and had therapeutic effects on ischemic brain injury when administered intranasally [86,87]. Since such research has technological value only when it is linked to clinical research, it is necessary to expand support for clinical translational research.

## 5. Exosome Application in Clinical Trials

**Figure 3:**
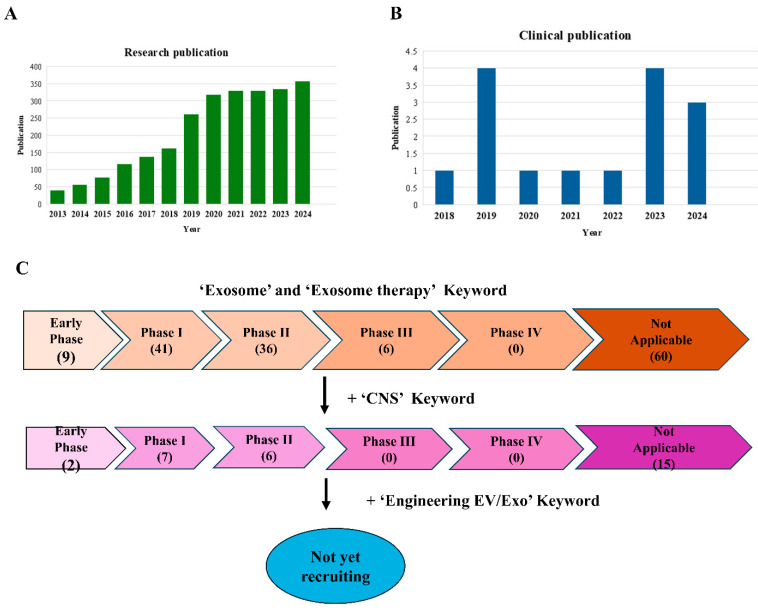
List of the exosome-based clinical trials conducted for therapy. (**A**) Recent years have seen a marked increase in the number of research publications focusing on exosome-based therapies. (**B**) The average number of clinical articles focusing on exosome-based therapies increases but remains limited. (**C**) The present study will compare the ClinicalTrials database with and without the Exosome keyword, as well as the ClinicalTrials database with and without the Exosome and CNS keywords.

The annual number of publications and citations reflects the research trends in a field. Exosomes are being studied as ideal candidates for the treatment of many diseases [26]. Many preclinical stages before clinical trials confirmed many benefits as a disease treatment [26]. In recent years, exosomes have been extensively investigated in clinical trials, and in particular, human-derived cell or sample-derived exosomes are being tried as therapeutic agents for various diseases. According to a survey on Pubmed NCBI (https://pubmed.ncbi.nlm.nih.gov) (accessed on 24 March 2025) and ClinicalTrials.gov (https://clinicaltrials.gov) (accessed on 24 March 2025), the core applications of exosomes are diagnostic biomarkers, exosome therapeutics, and clinical trials (Figure 3A,B). The number of research publications utilizing exosomes is increasing annually, with their potential for the treatment of CNS diseases being a prominent area of investigation. Nevertheless, the number of CNS clinical applications remains severely limited, and a substantial knowledge gap persists regarding the mechanisms and potential side effects of exosomal therapies before they can be safely deployed in clinical practice (Figure 3C). Exosome technology is still in the early stage of development, and there is no treatment available on the market in the global exosome treatment market yet because all therapeutics must maintain consistency and comparability for each batch produced [84]. In particular, since living cell therapy products or cell-derived exosome therapeutics are affected by cell conditions or microenvironments, managing the variation of the characteristic analysis values for each batch is the key to developing the therapy [88]. Cell-derived exosome samples are observed to vary in shape and size, with molecules distributed unevenly across the lipid membrane and within the exosome. This heterogeneity is an inherent characteristic of exosomes, and it is recognized as a significant challenge in the development of treatments that require consistency and equivalence [26].

Although exosome research has been identified as a potentially valuable preclinical treatment for CNS diseases, only a limited number of clinical trials have thus far confirmed the efficacy of exosome utilization as a treatment delivery vehicle for brain diseases [41,80]. To date, only endogenous exosomes have entered clinical trials, but brain-targeted engineered exosomes have yet to reach clinical translation, mainly due to large-scale production challenges and difficulties in uniform exosome production. Despite targeted engineering, EVs or exosomes (EVs/Exo) are still difficult to target to the brain, the pathogenic mechanism of CNS diseases, and determine their efficacy as therapeutics, as they are more likely to accumulate in the liver or kidneys. In addition, the possibility of unwanted cargo loading during the engineering process cannot be ruled out, which may not be beneficial for the treatment of CNS diseases. Nevertheless, a plethora of studies have demonstrated the efficacy of exosome-based therapy and diagnosis in various clinical trial stages. However, it is important to note that the majority of these clinical trials have been of a very limited scale about engineering EVs/Exo [89,90].

The manufacturing, isolation, purification, storage, and compliance of these limited engineered EVs/Exo in clinical trials must meet the high accuracy, safety, and regulatory requirements of a biopharmaceutical or gene therapy product. This necessitates management to ensure quality and efficacy [91,92]. Manufacturing involves the production of engineered viral vectors (adenoviruses and lentiviruses), typically using human or animal cell lines such as HEK293. The genetic material of a specific gene or modified virus is introduced into the cells, allowing them to multiply and produce large quantities of the vector. Culture conditions, yield, cell growth, and viral vector production must all be considered [93,94].

In the isolation process, the manufactured EV/Exo is extracted from cell cultures or tissues and sent to the purification step, where it is important to isolate the viral vector by either primary isolation of cells or filtration of cultures [6,95].

The subsequent stage is purification, which involves the separation of the harvested viral vector from impurities and its purification. This process can be achieved through various methodologies, including but not limited to centrifugation, chromatography, and filtration. However, it is imperative to emphasize that the paramount concern throughout this procedure is to guarantee the purity, activity, and safety of the vector [96]. In the context of clinical trials, the utilization of a safe and highly pure product is of paramount importance for the purpose of evaluation.

The storage step is the process of maintaining the stability of the final product, the EV/Exo vector, to preserve its quality. Storage considerations include temperature, safety, and freezing/thawing [97,98]. Given the sensitivity of EV/Exo vectors to temperature fluctuations during storage, it is imperative to identify an optimal storage temperature that aligns with the origin and engineering process of these vectors. Simultaneously, it is imperative to ensure the safety of the vector and to minimize damage incurred during the thawing process [99,100].

Finally, it is imperative to emphasize that regulatory compliance for the clinical phase is an ongoing process that must be meticulously prepared for at every stage. The manufacturing and culturing of EV/Exo vectors is a process that necessitates the assurance of the quality of the production environment and the adherence to Good Manufacturing Practice (GMP) standards. After this, Good Laboratory Practice (GLP) standards must be adhered to in order to ensure the reliability of the experiments and maintain the accuracy of the data. In the context of clinical trials, adherence to Good Clinical Practice (GCP) is imperative [26,101,102]. This encompasses experimental design, data collection and analysis, ethical considerations, and quality control and documentation that meets the stringent requirements of international regulatory agencies such as the U.S. FDA and European Medicines Agency (EMA) [103]. To date, no engineered EV/Exo has successfully progressed beyond this stage of clinical development, and there are numerous limitations that must be overcome.

However, recent advancements in global exosome research have led to the identification of heterogeneity and the characterization of new exosome types [93]. It is essential to develop an analytical method that will serve as a reference point for characterization [104,105]. The characterization method through single exosome analysis will be used in exosome research and industry in the future. It is expected to be of very high value as it can be applied in a variety of fields [106].

Moreover, research into the development of such technologies must address the limitations of existing technologies. Convergence research in engineering and bio is a potential solution to this issue; however, it requires support [107].

## 6. Conclusions

Recently, research is being actively conducted on procedures for creating engineered exosomes to target specific cells as a treatment for central nervous system diseases [108,109]. Exosomes deliver proteins and genetic information to recipient cells and are released as intercellular information carriers [110]. There are a variety of surface molecules fixed (Lamp-2, RGERPPR) to exosomes, allowing selective binding to specific recipient cells [111]. Therefore, engineering is needed to provide a route for exosomes to enter only specific cells and to distribute them intensively within in vivo tissues. Exosomes engineered in this way can diagnose certain central nervous system diseases at an early stage and can also lead to more positive treatment effects by understanding the mechanism of the disease and loading the exosomes with therapeutic agents [54,112]. However, background research still remains regarding the definition and safety limitations of exosomes, and diagnostic and therapeutic research using exosomes is aimed at establishing various platforms for future in vivo tracking, prognosis monitoring, and treatment amd must proceed actively [85].

Several papers claiming that exosomes carry miRNA need to be reassessed due to the possible presence of non-vesicular nanoparticles in the exosome samples [113].

In summary, as a CNS drug delivery tool, exosome properties differ from those of synthetic nanoparticles in a number of ways. These include low immunogenicity, high cargo-carrying capacity, and the ability to cross the blood-brain barrier [114,115,116]. In order to enhance the efficiency of CNS entry, it is possible to engineer the exosome surface to target the therapeutic target site and thus improve therapeutic efficacy. However, there is a paucity of research on the origin of engineered exosome, their structural stability, and how to improve their targeting efficiency.

Consequently, enhancing our comprehension of these deficient mechanisms and acknowledging their clinical potential with a focus on CNS diseases will facilitate the resolution of authentic patient concerns.

## Figures and Tables

**Table 1:** Summary of exosome administration routes in neurological diseases.

Disease	Donor Cell	Therapeutic Molecule	Intended Recipient Cells/Cargo	Drug Loading Method/Drug Route	Animal
Stroke	adipose-derived stem cells (ADSCs)	PEDF	unknown	Transfection/intravenous	Rats
	bone marrow mesenchymal stem cells (BMSC)	miR-124	Lamp2b-RVG	Electroporation/intravenous	Mice
	mesenchymal stem cells (MSCs)	miR-223-3p	unknown	Transfection/intravenous	Rats
Brain tumor	RAW 264.7	curcumin and SPIONs	RGE-peptide	Electroporation/intravenous	Mice
Spinal cord injury (SCI)	mesenchymal stem cells (MSC)	PTEN-siRNA	unknown	Co-incubation/intranasal	Rats
Parkinson’s disease	HEK-293T cells	Aptamer F5R1	Microglia, neurons, astrocytes	Co-incubation/intraperitoneal	Mice
Alzheimer’s disease	Astrocyte	miR-29	Neuron, Glia	Transfection/Intracerebral	Rats

## References

[1] Ni Z., Zhou S., Li S., Kuang L., Chen H., Luo X., Ouyang J., He M., Du X., Chen L. (2020). Exosomes: Roles and therapeutic potential in osteoarthritis. Bone Res..

[2] Beit-Yannai E., Tabak S., Stamer W.D. (2018). Physical exosome:exosome interactions. J. Cell. Mol. Med..

[3] Record M., Silvente-Poirot S., Poirot M., Wakelam M.J. (2018). Extracellular vesicles: Lipids as key components of their biogenesis and functions. J. Lipid Res..

[4] Doyle L., Wang M. (2019). Overview of Extracellular Vesicles, Their Origin, Composition, Purpose, and Methods for Exosome Isolation and Analysis. Cells.

[5] Mebarek S., Buchet R., Pikula S., Strzelecka-Kiliszek A., Brizuela L., Corti G., Collacchi F., Anghieri G., Magrini A., Ciancaglini P. (2023). Do Media Extracellular Vesicles and Extracellular Vesicles Bound to the Extracellular Matrix Represent Distinct Types of Vesicles?. Biomolecules.

[6] Zhang Y., Liu Y., Liu H., Tang W.H. (2019). Exosomes: Biogenesis, biologic function and clinical potential. Cell Biosci..

[7] Vizoso F.J., Eiro N., Cid S., Schneider J., Perez-Fernandez R. (2017). Mesenchymal Stem Cell Secretome: Toward Cell-Free Therapeutic Strategies in Regenerative Medicine. Int. J. Mol. Sci..

[8] Kolonics F., Szeifert V., Timár C.I., Ligeti E., Lőrincz Á.M. (2020). The Functional Heterogeneity of Neutrophil-Derived Extracellular Vesicles Reflects the Status of the Parent Cell. Cells.

[9] Zhang M., Hu S., Liu L., Dang P., Liu Y., Sun Z., Qiao B., Wang C. (2023). Engineered exosomes from different sources for cancer-targeted therapy. Signal Transduct. Target. Ther..

[10] Ratajczak K., Grel H., Olejnik P., Jakiela S., Stobiecka M. (2023). Current progress, strategy, and prospects of PD-1/PDL-1 immune checkpoint biosensing platforms for cancer diagnostics, therapy monitoring, and drug screening. Biosens. Bioelectron..

[11] Hazrati A., Mirsanei Z., Heidari N., Malekpour K., Rahmani-Kukia N., Abbasi A., Soudi S. (2023). The potential application of encapsulated exosomes: A new approach to increase exosomes therapeutic efficacy. Biomed. Pharmacother..

[12] Namiot E.D., Sokolov A.V., Chubarev V.N., Tarasov V.V., Schiöth H.B. (2023). Nanoparticles in Clinical Trials: Analysis of Clinical Trials, FDA Approvals and Use for COVID-19 Vaccines. Int. J. Mol. Sci..

[13] Bobo D., Robinson K.J., Islam J., Thurecht K.J., Corrie S.R. (2016). Nanoparticle-Based Medicines: A Review of FDA-Approved Materials and Clinical Trials to Date. Pharm. Res..

[14] Ezban M., Hermit M.B., Persson E. (2019). FIXing postinfusion monitoring: Assay experiences with N9-GP(nonacog beta pegol; Refixia^®^; Rebinyn^®^). Haemophilia.

[15] Hussen B.M., Faraj G.S.H., Rasul M.F., Hidayat H.J., Salihi A., Baniahmad A., Taheri M., Ghafouri-Frad S. (2022). Strategies to overcome the main challenges of the use of exosomes as drug carrier for cancer therapy. Cancer Cell Int..

[16] Alotaibi A.S., A Mahroos R., Al Yateem S.S., Menezes R.G. (2022). Central Nervous System Causes of Sudden Unexpected Death: A Comprehensive Review. Cureus.

[17] Charvériat M., Lafon V., Mouthon F., Zimmer L. (2021). Innovative approaches in CNS drug discovery. Therapies.

[18] Patel N.C. (2020). Methods to optimize CNS exposure of drug candidates. Bioorganic Med. Chem. Lett..

[19] Tonge P.J. (2018). Drug-Target Kinetics in Drug Discovery. ACS Chem. Neurosci..

[20] Gebeyehu A., Kommineni N., Meckes D.G., Sachdeva M.S. (2021). Role of Exosomes for Delivery of Chemotherapeutic Drugs. Crit. Rev. Ther. Drug Carr. Syst..

[21] Li Y., Chen J., Wang L., Lu M., Chopp M. (2001). Treatment of stroke in rat with intracarotid administration of marrow stromal cells. Neurology.

[22] Conaty P., Sherman L.S., Naaldijk Y., Ulrich H., Stolzing A., Rameshwar P. (2018). Methods of Mesenchymal Stem Cell Homing to the Blood-Brain Barrier. Methods Mol. Biol..

[23] Hade M.D., Suire C.N., Suo Z. (2021). Mesenchymal Stem Cell-Derived Exosomes: Applications in Regenerative Medicine. Cells.

[24] Alotaibi F. (2023). Exosomal microRNAs in cancer: Potential biomarkers and immunotherapeutic targets for immune checkpoint molecules. Front. Genet..

[25] Challagundla K.B., Wise P.M., Neviani P., Chava H., Murtadha M., Xu T., Kennedy R., Ivan C., Zhang X., Vannini I. (2015). Exosome-Mediated Transfer of microRNAs Within the Tumor Microenvironment and Neuroblastoma Resistance to Chemotherapy. JNCI J. Natl. Cancer Inst..

[26] Rezaie J., Feghhi M., Etemadi T. (2022). A review on exosomes application in clinical trials: Perspective, questions, and challenges. Cell Commun. Signal..

[27] Yang D., Zhang W., Zhang H., Zhang F., Chen L., Ma L., Larcher L.M., Chen S., Liu N., Zhao Q. (2020). Progress, opportunity, and perspective on exosome isolation—Efforts for efficient exosome-based theranostics. Theranostics.

[28] Jella K.K., Nasti T.H., Li Z., Malla S.R., Buchwald Z.S., Khan M.K. (2018). Exosomes, Their Biogenesis and Role in Inter-Cellular Communication, Tumor Microenvironment and Cancer Immunotherapy. Vaccines.

[29] Simons M., Raposo G. (2009). Exosomes—Vesicular carriers for intercellular communication. Curr. Opin. Cell Biol..

[30] He J., Ren W., Wang W., Han W., Jiang L., Zhang D., Guo M. (2022). Exosomal targeting and its potential clinical application. Drug Deliv. Transl. Res..

[31] Xu M., Feng T., Liu B., Qiu F., Xu Y., Zhao Y., Zheng Y. (2021). Engineered exosomes: Desirable target-tracking characteristics for cerebrovascular and neurodegenerative disease therapies. Theranostics.

[32] Parada N., Romero-Trujillo A., Georges N., Alcayaga-Miranda F. (2021). Camouflage strategies for therapeutic exosomes evasion from phagocytosis. J. Adv. Res..

[33] Lara P., Chan A.B., Cruz L.J., Quest A.F.G., Kogan M.J. (2020). Exploiting the Natural Properties of Extracellular Vesicles in Targeted Delivery towards Specific Cells and Tissues. Pharmaceutics.

[34] Kastelowitz N., Yin H. (2014). Exosomes and Microvesicles: Identification and Targeting By Particle Size and Lipid Chemical Probes. ChemBioChem.

[35] Van den Boorn J.G., Schlee M., Coch C., Hartmann G. (2011). SiRNA delivery with exosome nanoparticles. Nat. Biotechnol..

[36] Hood J.L. (2016). Post Isolation Modification of Exosomes for Nanomedicine Applications. Nanomedicine.

[37] Hornung S., Dutta S., Bitan G. (2020). CNS-Derived Blood Exosomes as a Promising Source of Biomarkers: Opportunities and Challenges. Front. Mol. Neurosci..

[38] Yim N., Ryu S.-W., Choi K., Lee K.R., Lee S., Choi H., Kim J., Shaker M.R., Sun W., Park J.-H. (2016). Exosome engineering for efficient intracellular delivery of soluble proteins using optically reversible protein–protein interaction module. Nat. Commun..

[39] Wang Y., Xiao T., Zhao C., Li G. (2023). The Regulation of Exosome Generation and Function in Physiological and Pathological Processes. Int. J. Mol. Sci..

[40] Lopes D., Lopes J., Pereira-Silva M., Peixoto D., Rabiee N., Veiga F., Moradi O., Guo Z.-H., Wang X.-D., Conde J. (2023). Bioengineered exosomal-membrane-camouflaged abiotic nanocarriers: Neurodegenerative diseases, tissue engineering and regenerative medicine. Mil. Med Res..

[41] Choi H., Choi K., Kim D.-H., Oh B.-K., Yim H., Jo S., Choi C. (2022). Strategies for Targeted Delivery of Exosomes to the Brain: Advantages and Challenges. Pharmaceutics.

[42] Huang L., Wu E., Liao J., Wei Z., Wang J., Chen Z. (2023). Research Advances of Engineered Exosomes as Drug Delivery Carrier. ACS Omega.

[43] Singh G., Mehra A., Arora S., Gugulothu D., Vora L.K., Prasad R., Khatri D.K. (2024). Exosome-mediated delivery and regulation in neurological disease progression. Int. J. Biol. Macromol..

[44] Kumari R., Jat P. (2021). Mechanisms of Cellular Senescence: Cell Cycle Arrest and Senescence Associated Secretory Phenotype. Front. Cell Dev. Biol..

[45] Fueller J., Herbst K., Meurer M., Gubicza K., Kurtulmus B., Knopf J.D., Kirrmaier D., Buchmuller B.C., Pereira G., Lemberg M.K. (2020). CRISPR-Cas12a–assisted PCR tagging of mammalian genes. J. Cell Biol..

[46] Hua Y., Dai X., Xu Y., Xing G., Liu H., Lu T., Chen Y., Zhang Y. (2022). Drug repositioning: Progress and challenges in drug discovery for various diseases. Eur. J. Med. Chem..

[47] Di Santo R., Niccolini B., Romanò S., Vaccaro M., Di Giacinto F., De Spirito M., Ciasca G. (2024). Advancements in Mid-Infrared spectroscopy of extracellular vesicles. Spectrochim. Acta Part A Mol. Biomol. Spectrosc..

[48] Théry C., Witwer K.W., Aikawa E., Alcaraz M.J., Anderson J.D., Andriantsitohaina R., Antoniou A., Arab T., Archer F., Atkin-Smith G.K. (2018). Minimal information for studies of extracellular vesicles 2018 (MISEV2018): A position statement of the International Society for Extracellular Vesicles and update of the MISEV2014 guidelines. J. Extracell. Vesicles.

[49] Migneault F., Dieudé M., Turgeon J., Beillevaire D., Hardy M.-P., Brodeur A., Thibodeau N., Perreault C., Hébert M.-J. (2020). Apoptotic exosome-like vesicles regulate endothelial gene expression, inflammatory signaling, and function through the NF-κB signaling pathway. Sci. Rep..

[50] Wang J., Jing J., Zhou C., Fan Y. (2024). Emerging roles of exosomes in oral diseases progression. Int. J. Oral Sci..

[51] Gurunathan S., Kang M.-H., Jeyaraj M., Qasim M., Kim J.-H. (2019). Review of the Isolation, Characterization, Biological Function, and Multifarious Therapeutic Approaches of Exosomes. Cells.

[52] Kim G.B., Shon O.-J., Seo M.-S., Choi Y., Park W.T., Lee G.W. (2021). Mesenchymal Stem Cell-Derived Exosomes and Their Therapeutic Potential for Osteoarthritis. Biology.

[53] Toh W.S., Foldager C.B., Pei M., Hui J.H.P. (2014). Advances in Mesenchymal Stem Cell-based Strategies for Cartilage Repair and Regeneration. Stem Cell Rev. Rep..

[54] Liang Y., Duan L., Lu J., Xia J. (2021). Engineering exosomes for targeted drug delivery. Theranostics.

[55] Luan X., Sansanaphongpricha K., Myers I., Chen H., Yuan H., Sun D. (2017). Engineering exosomes as refined biological nanoplatforms for drug delivery. Acta Pharmacol. Sin..

[56] Li S.-P., Lin Z.-X., Jiang X.-Y., Yu X.-Y. (2018). Exosomal cargo-loading and synthetic exosome-mimics as potential therapeutic tools. Acta Pharmacol. Sin..

[57] Tian T., Zhang H.-X., He C.-P., Fan S., Zhu Y.-L., Qi C., Huang N.-P., Xiao Z.-D., Lu Z.-H., Tannous B.A. (2018). Surface functionalized exosomes as targeted drug delivery vehicles for cerebral ischemia therapy. Biomaterials.

[58] Wang X., Wang X., Gong W., Gong W., Li R., Li R., Li L., Li L., Wang J., Wang J. (2024). Preparation of genetically or chemically engineered exosomes and their therapeutic effects in bone regeneration and anti-inflammation. Front. Bioeng. Biotechnol..

[59] Oshchepkova A., Zenkova M., Vlassov V. (2023). Extracellular Vesicles for Therapeutic Nucleic Acid Delivery: Loading Strategies and Challenges. Int. J. Mol. Sci..

[60] Lin T.-L., Lin Y.-H., Lee A.K.-X., Kuo T.-Y., Chen C.-Y., Chen K.-H., Chou Y.-T., Chen Y.-W., Shie M.-Y. (2023). The exosomal secretomes of mesenchymal stem cells extracted via 3D-printed lithium-doped calcium silicate scaffolds promote osteochondral regeneration. Mater. Today Bio.

[61] Sato Y.T., Umezaki K., Sawada S., Mukai S.-A., Sasaki Y., Harada N., Shiku H., Akiyoshi K. (2016). Engineering hybrid exosomes by membrane fusion with liposomes. Sci. Rep..

[62] Armstrong J.P.K., Holme M.N., Stevens M.M. (2017). Re-Engineering Extracellular Vesicles as Smart Nanoscale Therapeutics. ACS Nano.

[63] Xu B., Chen Y., Peng M., Zheng J.H., Zuo C. (2022). Exploring the potential of exosomes in diagnosis and drug delivery for pancreatic ductal adenocarcinoma. Int. J. Cancer.

[64] Fu S., Wang Y., Xia X., Zheng J.C. (2020). Exosome engineering: Current progress in cargo loading and targeted delivery. NanoImpact.

[65] Li Y., Gao Y., Gong C., Wang Z., Xia Q., Gu F., Hu C., Zhang L., Guo H., Gao S. (2018). A33 antibody-functionalized exosomes for targeted delivery of doxorubicin against colorectal cancer. Nanomedicine.

[66] Viñas J.L., Spence M., Gutsol A., Knoll W., Burger D., Zimpelmann J., Allan D.S., Burns K.D. (2018). Receptor-Ligand Interaction Mediates Targeting of Endothelial Colony Forming Cell-derived Exosomes to the Kidney after Ischemic Injury. Sci. Rep..

[67] Choi E.S., Song J., Kang Y.Y., Mok H. (2019). Mannose-Modified Serum Exosomes for the Elevated Uptake to Murine Dendritic Cells and Lymphatic Accumulation. Macromol. Biosci..

[68] Martín-Durán J.M., Hejnol A. (2021). A developmental perspective on the evolution of the nervous system. Dev. Biol..

[69] Zhong L., Wang J., Wang P., Liu X., Liu P., Cheng X., Cao L., Wu H., Chen J., Zhou L. (2023). Neural stem cell-derived exosomes and regeneration: Cell-free therapeutic strategies for traumatic brain injury. Stem Cell Res. Ther..

[70] Johnstone R.M. (1992). Maturation of reticulocytes: Formation of exosomes as a mechanism for shedding membrane proteins. Biochem. Cell Biol..

[71] Meng W., He C., Hao Y., Wang L., Li L., Zhu G. (2020). Prospects and challenges of extracellular vesicle-based drug delivery system: Considering cell source. Drug Deliv..

[72] Huo L., Du X., Li X., Liu S., Xu Y. (2021). The Emerging Role of Neural Cell-Derived Exosomes in Intercellular Communication in Health and Neurodegenerative Diseases. Front. Neurosci..

[73] Huang X., Ding J., Li Y., Liu W., Ji J., Wang H., Wang X. (2018). Exosomes derived from PEDF modified adipose-derived mesenchymal stem cells ameliorate cerebral ischemia-reperfusion injury by regulation of autophagy and apoptosis. Exp. Cell Res..

[74] Jeppesen D.K., Zhang Q., Franklin J.L., Coffey R.J. (2023). Extracellular vesicles and nanoparticles: Emerging complexities. Trends Cell Biol..

[75] Niazi S.K. (2023). Non-Invasive Drug Delivery across the Blood–Brain Barrier: A Prospective Analysis. Pharmaceutics.

[76] Jia G., Han Y., An Y., Ding Y., He C., Wang X., Tang Q. (2018). NRP-1 targeted and cargo-loaded exosomes facilitate simultaneous imaging and therapy of glioma in vitro and in vivo. Biomaterials.

[77] Laurent S., Forge D., Port M., Roch A., Robic C., Elst L.V., Muller R.N. (2008). Magnetic iron oxide nanoparticles: Synthesis, stabilization, vectorization, physicochemical characterizations, and biological applications. Chem. Rev..

[78] Du W., Feng Y., Wang X., Piao X., Cui Y., Chen L., Lei X., Sun X., Liu X., Wang H. (2013). Curcumin Suppresses Malignant Glioma Cells Growth and Induces Apoptosis by Inhibition of SHH/GLI1 Signaling Pathway in Vitro and Vivo. CNS Neurosci. Ther..

[79] Lee E.C., Ha T.W., Lee D.-H., Hong D.-Y., Park S.-W., Lee J.Y., Lee M.R., Oh J.S. (2022). Utility of Exosomes in Ischemic and Hemorrhagic Stroke Diagnosis and Treatment. Int. J. Mol. Sci..

[80] Ferguson S.W., Nguyen J. (2016). Exosomes as therapeutics: The implications of molecular composition and exosomal heterogeneity. J. Control Release.

[81] Lai J.J., Chau Z.L., Chen S., Hill J.J., Korpany K.V., Liang N., Lin L., Lin Y., Liu J.K., Liu Y. (2022). Exosome Processing and Characterization Approaches for Research and Technology Development. Adv. Sci..

[82] Fu J.M., Song W., Hao Z., Fan M.M., Li Y.M. (2023). Research trends and hotspots of exosomes in respiratory diseases. Medicine.

[83] Zhang Y., Bi J., Huang J., Tang Y., Du S., Li P. (2020). Exosome: A Review of Its Classification, Isolation Techniques, Storage, Diagnostic and Targeted Therapy Applications. Int. J. Nanomed..

[84] Yang X.-X., Sun C., Wang L., Guo X.-L. (2019). New insight into isolation, identification techniques and medical applications of exosomes. J. Control Release.

[85] Zou Z., Li H., Xu G., Hu Y., Zhang W., Tian K. (2023). Current Knowledge and Future Perspectives of Exosomes as Nanocarriers in Diagnosis and Treatment of Diseases. Int. J. Nanomed..

[86] Kim M., Lee Y., Lee M. (2021). Hypoxia-specific anti-RAGE exosomes for nose-to-brain delivery of anti-miR-181a oligonucleotide in an ischemic stroke model. Nanoscale.

[87] Guo S., Perets N., Betzer O., Ben-Shaul S., Sheinin A., Michaelevski I., Popovtzer R., Offen D., Levenberg S. (2019). Intranasal Delivery of Mesenchymal Stem Cell Derived Exosomes Loaded with Phosphatase and Tensin Homolog siRNA Repairs Complete Spinal Cord Injury. ACS Nano.

[88] Foo J.B., Looi Q.H., Chong P.P., Hassan N.H., Yeo G.E.C., Ng C.Y., Koh B., How C.W., Lee S.H., Law J.X. (2021). Comparing the Therapeutic Potential of Stem Cells and their Secretory Products in Regenerative Medicine. Stem Cells Int..

[89] Song J., Song B., Yuan L., Yang G. (2022). Multiplexed strategies toward clinical translation of extracellular vesicles. Theranostics.

[90] Kim H., Kim D., Nam H., Moon S., Kwon Y.J., Lee J.B. (2020). Engineered extracellular vesicles and their mimetics for clinical translation. Methods.

[91] Silva A.K., Morille M., Piffoux M., Arumugam S., Mauduit P., Larghero J., Bianchi A., Aubertin K., Blanc-Brude O., Noël D. (2021). Development of extracellular vesicle-based medicinal products: A position paper of the group “Extracellular Vesicle translatiOn to clinicaL perspectiVEs—EVOLVE France”. Adv. Drug Deliv. Rev..

[92] Brezgin S., Danilik O., Yudaeva A., Kachanov A., Kostyusheva A., Karandashov I., Ponomareva N., Zamyatnin A.A., Parodi A., Chulanov V. (2024). Basic Guide for Approaching Drug Delivery with Extracellular Vesicles. Int. J. Mol. Sci..

[93] Tan E., Chin C.S.H., Lim Z.F.S., Ng S.K. (2021). HEK293 Cell Line as a Platform to Produce Recombinant Proteins and Viral Vectors. Front. Bioeng. Biotechnol..

[94] Lee C.S., Bishop E.S., Zhang R., Yu X., Farina E.M., Yan S., Zhao C., Zeng Z., Shu Y., Wu X. (2017). Adenovirus-mediated gene delivery: Potential applications for gene and cell-based therapies in the new era of personalized medicine. Genes Dis..

[95] Konoshenko M.Y., Lekchnov E.A., Vlassov A.V., Laktionov P.P. (2018). Isolation of Extracellular Vesicles: General Methodologies and Latest Trends. BioMed Res. Int..

[96] Minh A.D., Kamen A.A. (2021). Critical Assessment of Purification and Analytical Technologies for Enveloped Viral Vector and Vaccine Processing and Their Current Limitations in Resolving Co-Expressed Extracellular Vesicles. Vaccines.

[97] Kusuma G.D., Barabadi M., Tan J.L., Morton D.A.V., Frith J.E., Lim R. (2018). To Protect and to Preserve: Novel Preservation Strategies for Extracellular Vesicles. Front. Pharmacol..

[98] Welsh J.A., Goberdhan D.C.I., O’Driscoll L., Buzas E.I., Blenkiron C., Bussolati B., Cai H., Di Vizio D., Driedonks T.A.P., Erdbrügger U. (2024). Minimal information for studies of extracellular vesicles (MISEV2023): From basic to advanced approaches. J. Extracell. Vesicles.

[99] Zeng H., Guo S., Ren X., Wu Z., Liu S., Yao X. (2023). Current Strategies for Exosome Cargo Loading and Targeting Delivery. Cells.

[100] Maroto R., Zhao Y., Jamaluddin M., Popov V.L., Wang H., Kalubowilage M., Zhang Y., Luisi J., Sun H., Culbertson C.T. (2017). Effects of storage temperature on airway exosome integrity for diagnostic and functional analyses. J. Extracell. Vesicles.

[101] Muthu S., Bapat A., Jain R., Jeyaraman N., Jeyaraman M. (2021). Exosomal therapy—A new frontier in regenerative medicine. Stem Cell Investig..

[102] Harn H.-J., Chen Y.-S., Lin E.-Y., Chiou T.-W. (2020). Exosomes in clinical trial and their production in compliance with good manufacturing practice. Tzu Chi Med. J..

[103] Wang C., Tsai T., Lee C. (2024). Regulation of exosomes as biologic medicines: Regulatory challenges faced in exosome development and manufacturing processes. Clin. Transl. Sci..

[104] Liu W.-Z., Ma Z.-J., Kang X.-W. (2022). Current status and outlook of advances in exosome isolation. Anal. Bioanal. Chem..

[105] Wang X., Tian L., Lu J., Ng I.O.-L. (2022). Exosomes and cancer—Diagnostic and prognostic biomarkers and therapeutic vehicle. Oncogenesis.

[106] Miron R.J., Zhang Y. (2024). Understanding exosomes: Part 1-Characterization, quantification and isolation techniques. Periodontol. 2000.

[107] Li X., Corbett A.L., Taatizadeh E., Tasnim N., Little J.P., Garnis C., Daugaard M., Guns E., Hoorfar M., Li I.T.S. (2019). Challenges and opportunities in exosome research-Perspectives from biology, engineering, and cancer therapy. APL Bioeng..

[108] Wang W., Sun H., Duan H., Sheng G., Tian N., Liu D., Sun Z. (2024). Isolation and usage of exosomes in central nervous system diseases. CNS Neurosci. Ther..

[109] Khan S.U., Khan M.I., Khan M.U., Khan N.M., Bungau S., Hassan S.S.U. (2022). Applications of Extracellular Vesicles in Nervous System Disorders: An Overview of Recent Advances. Bioengineering.

[110] Butreddy A., Kommineni N., Dudhipala N. (2021). Exosomes as Naturally Occurring Vehicles for Delivery of Biopharmaceuticals: Insights from Drug Delivery to Clinical Perspectives. Nanomaterials.

[111] Isaac R., Reis F.C.G., Ying W., Olefsky J.M. (2021). Exosomes as mediators of intercellular crosstalk in metabolism. Cell Metab..

[112] Li Q., Fu X., Kou Y., Han N. (2023). Engineering strategies and optimized delivery of exosomes for theranostic application in nerve tissue. Theranostics.

[113] Herrmann I.K., Wood M.J.A., Fuhrmann G. (2021). Extracellular vesicles as a next-generation drug delivery platform. Nat. Nanotechnol..

[114] Bunggulawa E.J., Wang W., Yin T., Wang N., Durkan C., Wang Y., Wang G. (2018). Recent advancements in the use of exosomes as drug delivery systems. J. Nanobiotechnol..

[115] EL Andaloussi S., Lakhal S., Mäger I., Wood M.J. (2013). Exosomes for targeted siRNA delivery across biological barriers. Adv. Drug Deliv. Rev..

[116] Ingato D., Lee J.U., Sim S.J., Kwon Y.J. (2016). Good things come in small packages: Overcoming challenges to harness extracellular vesicles for therapeutic delivery. J. Control Release.

